# Trunk Fat Negatively Influences Skeletal and Testicular Functions in Obese Men: Clinical Implications for the Aging Male

**DOI:** 10.1155/2013/182753

**Published:** 2013-11-20

**Authors:** Silvia Migliaccio, Davide Francomano, Roberto Bruzziches, Emanuela A. Greco, Rachele Fornari, Lorenzo M. Donini, Andrea Lenzi, Antonio Aversa

**Affiliations:** ^1^Department of Movement, Human and Health Sciences, Unit of Endocrinology, University of Rome “Foro Italico”, Largo Lauro De Bosis 15, 00195 Rome, Italy; ^2^Department of Experimental Medicine, Medical Pathophysiology, Food and Science and Endocrinology Section, “Sapienza” University of Rome, Viale Regina Elena 324, 00161 Rome, Italy

## Abstract

Osteocalcin (OSCA) seems to act as a negative regulator of energy metabolism and insulin sensitivity. Evidence from male rodents suggests that OSCA may also regulate testosterone (T) synthesis. Using a cross-sectional design, we evaluated OSCA, 25(OH) vitamin D, T, 17**β**-estradiol (E2), homeostasis model assessment of insulin resistance (HOMA-IR), and body composition in 86 obese (mean BMI = 34) male subjects (18–69 yr old). Independently from BMI, an inverse relationship between trunk fat percentage and plasma T (*r* ^2^ = −0.26, *P* < 0.01) and between HOMA-IR and OSCA levels (*r* ^2^ = −0.22, *P* < 0.005) was found. OSCA levels, as well as vitamin D, decreased significantly for higher BMI with significant differences above 35 (*P* < 0.01). A direct correlation between T and bone mineral density at lumbar (BMDL) and neck (BMDH) (*P* < 0.001, *r* ^2^ = −0.20; *P* < 0.001, *r* ^2^ = −0.24) was found, independently from age. An inverse correlation between E2 levels, BMDL, and BMDH (*P* < 0.001, *r* ^2^ = −0.20; *P* < 0.001, *r* ^2^ = −0.19) was observed. These data provide new evidences that a relationship between trunk fat mass, insulin sensitivity, OSCA and T synthesis occurs. This new relationship with skeletal health has relevant implications for the aging male, suggesting OSCA as a novel marker of metabolic and gonadal health status.

## 1. Introduction

Emerging data suggest that bone mass, energy metabolism, and reproduction may be coordinately regulated. It is now accepted that bone is an endocrine organ favouring whole-body glucose homeostasis and energy expenditure. These functions of bone are, at least in part, mediated by an osteoblast-specific secreted molecule, osteocalcin (OSCA), that, when uncarboxylated (ucOSCA), acts as a hormone favouring *β* cell proliferation, insulin secretion and sensitivity, and energy expenditure. Also, the recent study by Oury et al. reveals that, in rodents, the bone is a positive regulator of male fertility, and that this action may be mediated through OSCA, via binding to a specific receptor present on Leydig cells that favours testosterone (T) biosynthesis [1]. OSCA-deficient mice show a decrease in testicular, epididymal, and seminal vesicles weights and sperm count, and Leydig cell maturation appears to be halted in absence of OSCA. Those results, along with others previously published, support the hypothesis that regulations of bone remodelling, energy metabolism, and reproduction are linked [2, 3]. 

Ageing decreases circulating levels of sex steroid hormones in men [4, 5], and it is associated with visceral fat accumulation at the abdominal level. Male obesity might be associated with a reduction of T levels, as well as with sexual disturbances [6]. At present, the effects of raising T levels on bone mineral density (BMD) in men with metabolic syndrome (central obesity) have been investigated by our group demonstrating that a 5%/year increase in bone mineral density occurs in this population. However, in that previous study, OSCA levels were not evaluated [7].

Thus, the aim of the present study was to evaluate the potential relationship between circulating levels of OSCA and T with adipose tissue and BMD in obese men.

## 2. Materials and Methods

### 2.1. Subjects

A cross-sectional study was made in 86 consecutive outclinic male adult subjects. Physical examination and routine biochemistry were performed to exclude significant diseases. Some of the obese subjects had an impaired glucose tolerance test (according to WHO criteria), but none was overtly diabetic [8]. Some had moderate hypertension. None of the subjects had modified prior medications and body weight over six months or reported excessive alcohol consumption before clinical investigation. Smokers were not considered as a separate group. Exclusion criteria were chronic medical conditions, vitamin D supplementation, or the use of medications affecting bone metabolism, hormonal and nutritional status, recent weight loss, and prior bariatric surgery interventions. All subjects provided informed consent before taking part in the study, and the local ethical committee approved the research protocol.

### 2.2. Study Protocol

Body mass index (BMI) was calculated dividing weight (kilograms) by the square of length (meters). Blood samples were obtained in the morning (07.00-08.00 am) after an overnight fast. Sera were frozen at −80°C until analysis. All subjects underwent an oral glucose (75 g) tolerance test, and samples were taken at 0, 30, 60, 90, 120, and 180 minutes for glucose and insulin determinations, as well as routine assay for total and HDL cholesterol and triglycerides. To assess insulin sensitivity, we calculated the HOMA-IR using the formula (fasting insulin in mU/L × fasting glucose in mmol/L)/22.5. The hormonal evaluation included OSCA, 17*β*-estradiol (E2), T, 25-OH vitamin D (vitamin D), parathyroid hormone (PTH), sex hormone binding globulin (SHBG), thyroid stimulating hormone (TSH), free T3 (fT3), free T4 (fT4), and prolactin (PRL). Nonspecific inflammatory markers as fibrinogen and C reactive protein (CRP) were also evaluated. 

### 2.3. Assays

OSCA, T, E2, TSH, fT3, and fT4 were measured with solid phase commercial RIAs (provided by Diagnostic Products, Los Angeles, CA, and Diagnostics Systems Laboratories, Inc., Webster, TX). TSH, PRL, PTH, and SHBG levels were measured by immunoradiometric assay (provided by Diagnostic Products, Diagnostics Systems Laboratories, Inc., and Radim, Pomezia, Italy). Plasma glucose, serum total cholesterol, HDL cholesterol, and triglycerides were measured by an automated clinical chemistry analyser (Modular P, Roche Diagnostics GmbH, Mannheim, Germany). Insulin and vitamin D levels were measured by radioimmunoassay while CRP circulating levels were measured by latex agglutination. The intra- and interassay coefficients of variations for all hormonal assays ranged between 3.4–6.2% and 3.6–8.4%, respectively. All determinations were performed in duplicate. 

### 2.4. Measurements

Anthropometric measurements included weight and height; body weight was measured as the subjects were fasting overnight and wearing underwear. Body fat mass, fat-free mass (kg), and both lumbar and femoral BMD were measured by dual-energy X-ray absorptiometry (DEXA) (Hologic 4500 RDR), with coefficient of variation of <1% for bone density and <1.5% for fat mass [9]. Amount of trunk fat mass was distinguished from peripheral and appendicular fat mass as a measure of abdominal adiposity. In particular, trunk fat was defined as the adipose tissue localized within the region below the chin, delineated by vertical lines within the left and right glenoid fossae bordering laterally to the ribs and by the oblique lines that cross the femoral necks and converge below the pubic symphysis [10]. 

### 2.5. Statistical Analysis

Data are presented as the mean ± SD of absolute value except for skewed variables, which were presented as median (interquartile range 25–75%). Continuous variables were normally distributed (Shapiro-Wilk test) and were analysed using Student's *t*-test for paired or unpaired data, Pearson's *χ* ^2^ test, Wilcoxon's signed-rank test, and Spearman's correlation analysis, as appropriate. Multiple stepwise regression analysis was performed to determine the associations between serum OSCA, BMD, T, and E2 concentration after adjusting for potential confounders. A *P* value < 0.05 ± SD was considered statistically significant. Statistical analysis was performed using the computer statistical package SPSS/10.0 (SPSS, Chicago, IL, USA) and SAS/6.4 (SAS Institute Cary, NC, USA).

## 3. Results

Baseline characteristics of the study population are shown in Table 1. Eighty-six adult men (mean age 45 yrs) were subdivided, according to their BMI, into overweight (BMI < 30), class-I obesity (BMI > 30 ≤ 35), class-II obesity (BMI > 35 ≤ 40), and class-III obesity (BMI > 40). Each group showed normal levels of total and HDL cholesterol and triglycerides (Table 1). No significant difference in the percentage of smokers and hypertensive men among the groups was present. Increased HOMA index (*P* < 0.0001), plasma fibrinogen, and C reactive protein (*P* < 0.0001) but lower levels of vitamin D (*P* < 0.0001) were found (Table 1). As expected, both trunk fat and HOMA increased for higher BMIs (*P* < 0.0001, resp.); regression analysis demonstrated that trunk fat was found to be the independent variable from BMI (Table 2).

**Figure 1:**
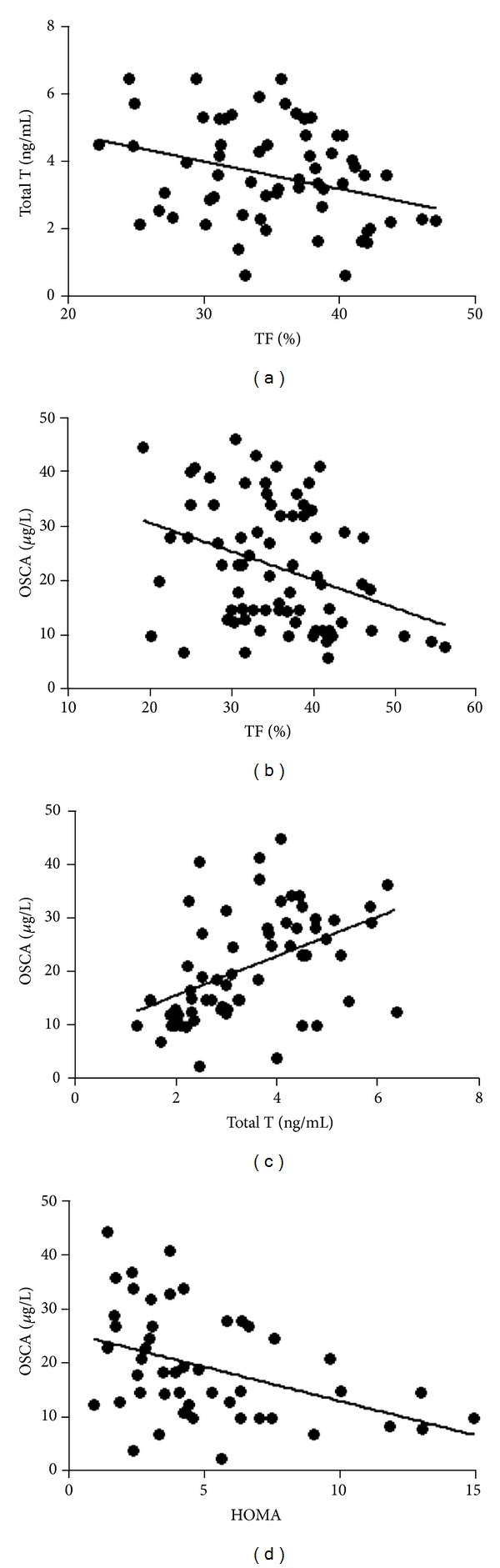
Correlation between (a) total testosterone (total T) and trunk fat (TF) (*P* < 0.01, *r* ^2^ = −0.26), (b) osteocalcin (OSCA) and TF (*P* < 0.001, *r* ^2^ = −0.17), (c) OSCA and Total T (*P* < 0.0001, *r* ^2^ = 0.23), (d) OSCA and HOMA (*P* < 0.001, *r* ^2^ = −0.20).

**Figure 2:**
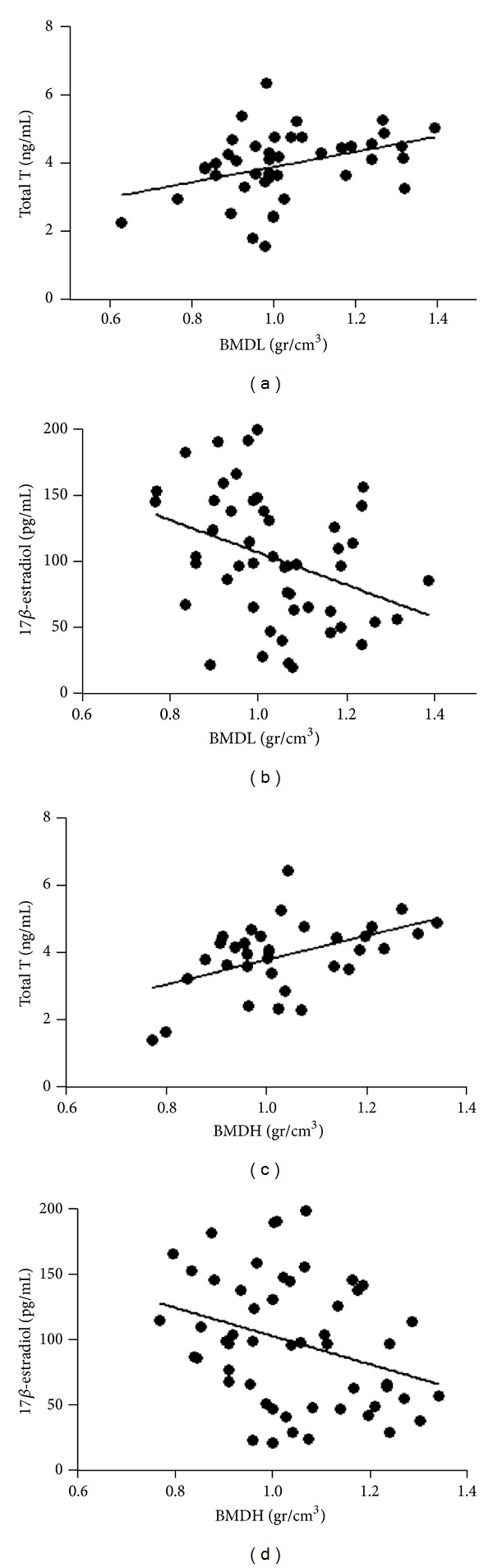
Correlation between (a) total testosterone (total T) and lumbar bone mineral density (BMDL) (*P* < 0.001, *r* ^2^ = −0.20), (b) 17*β*-estradiol and BMDL (*P* < 0.001, *r* ^2^ = −0.20), (c) total T and hip BMD (BMDH) (*P* < 0.001, *r* ^2^ = −0.24), (d) 17*β*-estradiol and BMDH (*P* < 0.001, *r* ^2^ = −0.19).

T levels were evaluated in different BMI subgroups, and, as expected, they were significantly lower in the higher BMI categories (BMI > 35 ≤ 40, *P* < 0.01; BMI > 40, *P* < 0.001; Table 1) but independent of age. Also, T showed an inverse relationship with trunk fat (*P* < 0.01, *r* ^2^ = −0.26; Figure 1(a)) but a direct relationship with OSCA (*P* < 0.0001, *r* ^2^ = 0.23; Figure 1(c)). Noteworthy, OSCA levels showed the same trend to decrease in the groups with a higher BMI (BMI 35 ≤ 40, *P* < 0.01; BMI > 40, *P* < 0.0001; Table 1) showing also an inverse relationship with HOMA index (*P* < 0.001, *r* ^2^ = −0.20; Figure 1(d)) and trunk fat (*P* < 0.001, *r* ^2^ = −0.17; Figure 1(b)). A direct correlation between T and bone mineral density at lumbar (BMDL) and neck hip site (BMDH) (*P* < 0.001, *r* ^2^ = −0.20, *P* < 0.001Figure 2(a); *r* ^2^ = −0.24, Figure 2(c), respectively) was found; regression analysis showed that T levels were independent from age (Table 2(b)). On the contrary, an inverse correlation between 17*β*-estradiol (E2) serum levels and BMDL and BMDH (*P* < 0.001, *r* ^2^ = −0.20, *P* < 0.001Figure 2(b); *r* ^2^ = −0.19, Figure 2(d), respectively) was observed. 

## 4. Discussion

As far as we are aware, this is the first study that demonstrates a relationship between metabolic, bone, and testicular functions in humans. In particular, our retrospective analysis carried out in a series of overweight and obese outclinic patients shows that OSCA, a product protein produced by osteoblasts [11], involved in multiple regulatory pathways, might play a pivotal role in the regulation of glucose metabolism, energy expenditure, and testosterone synthesis in humans. Trunk-fat mass influences cardiovascular diseases because of its impact on glucose and lipid metabolism [12, 13].

Elevated OSCA levels have been associated with improved glucose tolerance and with increased *β* cell function and insulin sensitivity [14]. Indeed, the uncarboxylated forms of OSCA (ucOSCA) appear to be associated with improved glucose tolerance in healthy men [15]. Thus, the balance between cOSCA and ucOSCA seems to be a key factor in this paradigm. Alfadda et al. [16] found a relationship between OSCA and lipid indices in patients with T2DM, and both OSCA and ucOSCA were significantly lower in patients with metabolic syndrome (MetS) compared to those without MetS, independently of BMI. In patients with MetS, ucOSCA was significantly and positively correlated with HDL cholesterol, while OSCA was significantly and negatively correlated with serum triglycerides [16, 17].

In the present study, we did not investigate whether this carboxylation plays an active role in biological actions of OSCA. However, it is known that circulating OSCA concentration is associated with parameters of glucose metabolism, insulin sensitivity, and fat mass in humans [18]. These observations clearly suggest a role of OSCA as a regulator of systemic energy metabolism so that we can speculate that the skeleton might act as an endocrine organ by secreting OSCA, which leads to increased insulin secretion, lowering blood glucose, and increasing insulin sensitivity and energy expenditure. The endocrine interplay between insulin, osteoblast, and OSCA seems to represent a complex regulatory pathway. In this loop, we were able to demonstrate that additional components may be added and that OSCA may represent a positive regulator of T production. Additionally, our data show that our patients were vitamin D deficient according to their BMI, and, thus, vitamin D deficiency might have also played a role in the reduced T levels. Interestingly, trunk fat more than BMI was an independent predictor factor of vitamin D levels, and, furthermore, reduced T levels resulted to be independent of age. It must be pointed out that, as previously shown by others [16], we have not found significant correlation between E2 levels and bone mineral density at both lumbar and femoral sites. Indeed, previous studies have suggested a pivotal role of estrogens in the regulation of skeletal homeostasis in men [19, 20]. However, our data do not support a correlation between E2 levels and higher bone mineral density and are in agreement with recent findings on the positive regulatory role of E2 in body composition and sexual function in men [21]. On the contrary, lower levels of T significantly correlate with lower bone mineral density in obese male. These data strongly indicate that androgens, more than estrogens, play a pivotal role in the maintenance of male skeletal health. 

 Finally, an alteration of vitamin D levels and low OSCA level altered insulin sensitivity strongly suggesting the existence of an important interplay between bone tissue, energy metabolism, and gonadal status, likely for the presence of a common pathogenic mechanism leading to the development of metabolic and skeletal diseases. 

## 5. Conclusions

Our data provide, for the first time, new lines of evidence of the role of OSCA. In fact, a relationship between visceral fat mass (not BMI), insulin sensitivity, OSCA, and testosterone synthesis occurs in humans, which significantly correlates with skeletal health. Furthermore, OSCA may exert different actions on metabolic and gonadal health status, other than the well-established function as marker of bone remodelling. In our view, these findings have relevant implications for the ageing male in that they clearly suggest OSCA as a novel marker for metabolic, skeletal, and testicular health throughout the life. 

## Figures and Tables

**Table 1:** Biochemical and hormonal characteristics of the patients according to different BMI. Values are expressed as means ± SD.

	BMI < 30 (*n* = 20)	BMI 30 ≤ 35 (*n* = 22)	BMI 35 ≤ 40 (*n* = 22)	BMI > 40 (*n* = 22)	
BMI	27 ± 1.2	32 ± 1.6**	38 ± 1.5***	44 ± 4***	**P < 0.001 ***P < 0.0001
Mean age (yrs)	51 ± 12	42 ± 18	47 ± 10	46 ± 13	—
Total chol. (mg/dL)	195 ± 43	197 ± 42	215 ± 34	202 ± 39	—
HDL (mg/dL)	42 ± 10	42 ± 7	41 ± 6	44 ± 4	—
TGL (mg/dL)	119 ± 53	131 ± 54	126 ± 52	151 ± 58	—
Fibrinogen (mg/dL)	263 ± 81	303 ± 70	366 ± 76	402 ± 133**	**P < 0.001
CRP (ng/mL)	3 ± 1.5	3 ± 1.8	4 ± 2.4	5.5 ± 2.1**	**P < 0.001
HOMA	2.7 ± 1.4	4.4 ± 2.1***	5.7 ± 2.5***	6.4 ± 2.2***	***P < 0.0001
OSCA (*µ*g/L)	25.34 ± 10.7	19 ± 10.1	16.6 ± 9.1*	12.5 ± 7.1***	*P < 0.01 ***P < 0.0001
SHBG (nmol/L)	25 ± 10	24 ± 13	25 ± 10	27 ± 12	—
PTH (pg/mL)	38 ± 14	39 ± 13	39 ± 13	49 ± 14	—
Vitamin D (ng/mL)	29 ± 7	23 ± 5*	19 ± 8**	14 ± 9***	*P < 0.01 **P < 0.001 ***P < 0.0001
17*β*-E2 (pg/mL)	29 ± 11	32 ± 9	31 ± 13	35 ± 16	—
Testosterone (ng/mL)	4.4 ± 1.06	4.5 ± 1.24	3.17 ± 1.32*	2.69 ± 1.1**	*P < 0.01 **P < 0.001
Trunk fat (%)	25.5 ± 6.6	33.74 ± 6.1***	37.11 ± 5.2***	41.86 ± 7.1***	***P < 0.0001

**Table:** (a)

Model		Coefficients^a^			*t*	Siq.
		Unstandardized coefficients		Standardized coefficients		
		*B*	Std. error	Beta		
1	(Constant)	25.611	5.582		4.588	0.000
	OSCA	0.015	0.080	0.027	0.185	0.854
	HOMA	−0.130	0.297	−0.069	−0.437	0.665
	Total_T	0.492	0.499	0.147	0.985	0.330
	FBN	−0.002	0.008	−0.036	−0.208	0.836
	PCR	−0.329	0.332	−0.154	−0.990	0.328
	Trunk_Fat	0.224	0.100	0.330	2.253	0.030

^a^Dependent variable: BMI.

**Table:** (b)

Model		Coefficients^a^			*t*	Siq.
		Unstandardized coefficients		Standardized coefficients		
		*B*	Std. error	Beta		

1	(Constant)	71.519	15.074		4,745	0.000
	OSCA	−0.098	0.216	−0.068	−0.454	0.652
	HOMA	−0.441	0.802	−0.088	−0.550	0.585
	Total_T	−3.447	1.348	−0.387	−2.558	0.014
	FBN	0.006	0.023	0.043	0.244	0.808
	PCR	0.271	0.897	0.048	0.302	0.764
	Trunk_Fat	−0.290	0.269	−0.160	−1.078	0.288

^a^Dependent variable: Age.
